# Cool but not harmless: synthetic cooling agents enhance nicotine reinforcement via the mesolimbic dopamine system

**DOI:** 10.1038/s41386-026-02503-1

**Published:** 2026-07-23

**Authors:** Mary Amir Bishara, Giordano de Guglielmo

**Affiliations:** Department of Psychiatry, University of California San Diego, La Jolla, CA USA

**Keywords:** Addiction, Risk factors

For decades, menthol has held a special status in tobacco regulation. Its cooling sensation makes inhaled nicotine smoother and easier to tolerate, and a large literature links menthol to heavier use and greater difficulty quitting. As U.S. jurisdictions have moved to restrict menthol in combustible and electronic nicotine products, however, the market has not gone flavorless. Manufacturers have instead adopted synthetic cooling agents—most prominently WS-3 and WS-23—that share menthol’s agonism at the cold-sensing TRPM8 receptor, reproducing its cooling sensation without the minty taste. These compounds now appear widely in “non-menthol” and “ice” e-cigarette products, in some cases at concentrations higher than menthol itself, yet they remain unregulated for inhalation [1]. The pressing question is whether synthetic coolants are inert sensory substitutes or whether, like menthol, they increase the abuse liability of nicotine products.

That question is not idle, because menthol is far from a passive flavorant. Work over the past decade has shown that menthol enhances nicotine reward and reinforcement through specific neurobiological actions: it behaves as a chemical chaperone that upregulates nicotinic acetylcholine receptors (nAChRs), increases the intrinsic excitability of ventral tegmental area (VTA) dopamine neurons, and augments dopamine release in the nucleus accumbens (NAc) [2]. Behaviorally, menthol increases nicotine self-administration in rodents [3], and these molecular and circuit actions offer a plausible substrate for that effect. If chemically distinct coolants were to converge on these same nodes, it would imply that moving away from menthol has done little to lower nicotine’s addictive potential of the products that replaced it.

In this issue, Maddox, Henderson, and colleagues provide a direct test [4]. Using an e-Vape self-administration model in male and female mice, they show that pairing nicotine with a WS-3/WS-23 mixture significantly increases the number of earned vapor deliveries beyond nicotine alone, to a degree comparable with menthol. The behavioral effect tracks with changes in dopamine signaling measured two complementary ways. Fiber photometry of the dopamine sensor dLight1.3b in the NAc core showed that the coolants amplified both cue-evoked and intake-evoked dopamine transients, and fast-scan cyclic voltammetry in brain slices showed greater electrically evoked dopamine release under both tonic and phasic stimulation, with the coolant group again significantly exceeding nicotine alone.

At the cellular level, whole-cell recordings from VTA dopamine neurons showed that coolant exposure lowered rheobase and raised firing—indicating a rise in intrinsic excitability of the dopamine neurons. The authors also recorded neighboring VTA GABA neurons, which normally provide inhibitory tone onto dopamine cells: acute nicotine sharply reduced their firing, and this disinhibition offers a complementary route by which the same exposure can raise dopamine-neuron output. Receptor imaging completed the picture, showing enhanced upregulation of α4- and α6-containing nAChRs (α4* and α6*). Importantly, a coolant-only group that received no nicotine failed to discriminate active from inactive responses and produced little intake-evoked dopamine, indicating that the coolants are not reinforcing on their own but instead potentiate the actions of nicotine.

**Fig. 1:**
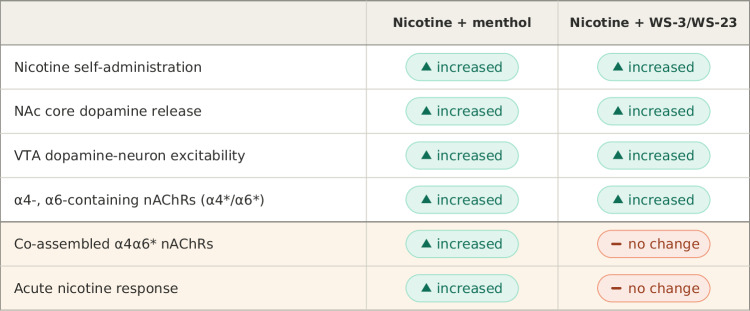
Synthetic coolants mirror menthol across the mesolimbic dopamine system, diverging only at the co-assembled α4α6* nicotinic receptor. Comparison of nicotine + menthol and nicotine + WS-3/WS-23. Relative to nicotine alone, both increase nicotine self-administration, cue- and intake-evoked dopamine release in the nucleus accumbens (NAc) core, the intrinsic excitability of ventral tegmental area (VTA) dopamine neurons, and the overall pools of α4- and α6-containing nicotinic acetylcholine receptors (nAChRs; α4* and α6*). The two diverge at the high-sensitivity subtype in which α4 and α6 co-assemble in the same pentamer (α4α6*): menthol upregulates α4α6* and potentiates the acute nicotine response, whereas WS-3/WS-23 leave both unchanged. Without nicotine, the coolants are not independently reinforcing. “Increased” is relative to nicotine alone; α4*/α6* denote the broad α4- and α6-containing pools and α4α6* the co-assembled subset. Coolant effects and the behavioral and dopamine comparisons are from Maddox et al.; menthol’s excitability and receptor-subtype profile is established from earlier work (ref. [2]). DA dopamine, GABA γ-aminobutyric acid, nAChR nicotinic acetylcholine receptor, NAc nucleus accumbens, VTA ventral tegmental area.

The most informative part of the study, to our reading, is where coolants and menthol diverge (Fig. 1). Although WS-3/WS-23 reproduced menthol’s enhancement of behavior, dopamine release, and excitability, their effect on nicotinic receptors was subtype-selective. The coolants raised the overall α4- and α6-containing pools, with the α4 pool rising even above nicotine alone, but they did not increase the specific subpopulation in which α4 and α6 co-assemble in the same pentamer—α4α6* receptors. That subpopulation matters because it is the high-sensitivity subtype most closely tied to the acute response to smoking-relevant nicotine [5].

Consistent with that gap, acute nicotine application produced the same roughly three-fold increase in dopamine-neuron firing regardless of prior coolant exposure: the coolants raised baseline excitability but did not further potentiate the acute nicotine response, in contrast to menthol. Notably, the GABAergic disinhibition described above was common to all nicotine-containing conditions, so it cannot by itself explain the coolant-specific enhancement of nicotine reinforcement.

The authors interpret these results sensibly as evidence that the two additive classes share much of their mechanism without being identical. The α4α6* difference rests on a null result and would be worth confirming directly, though if it holds it points to a genuine pharmacological distinction between additives now treated as interchangeable. Either way, the data reframe synthetic coolants as active modulators of the mesolimbic dopamine system rather than neutral stand-ins for menthol.

The translational message is clear and timely. The results of the study of Maddox et al. indicate that products marketed as “non-menthol” are not free of the pharmacological effects that drew regulatory scrutiny to menthol, and the abuse-liability assessment applied to menthol should extend to synthetic coolants. The stakes are highest for young people, among whom flavored and “ice” products are disproportionately popular and often serve as an entry point to regular nicotine use; a framework that removes menthol while leaving pharmacologically active coolants untouched may simply redirect, rather than reduce, that risk.

Finally, several questions remain. WS-3 and WS-23 were studied only as a fixed combination, leaving their individual contributions and any synergy unresolved. The causal chain linking nAChR upregulation, dopamine-neuron excitability, and behavior remains to be tested directly. And the consequences of chronic, escalating exposure—the pattern most relevant to human vapers—are still unknown. By bringing behavior, neurochemistry, and physiology together on a fast-moving regulatory problem, Maddox and colleagues make a compelling case that the chemistry of “cool” warrants the same scrutiny we have learned to apply to menthol.
